# What is the prevalence of overactive bladder symptoms in a lower socioeconomic female population? A suggestion for a study in India

**DOI:** 10.4103/0970-1591.32074

**Published:** 2007

**Authors:** Kaytan V. Amrute, Brian Vanderbrink, Gopal H. Badlani

**Affiliations:** Department of Ob/Gyn, Woodhull Medical Center, Brooklyn, NY, USA; * Department of Urology, Long Island Jewish Center, New Hyde Park, NY, USA; ** Department of Urology, Wake Forest University Baptist Medical Center, Winston-Salem, NC, USA

**Keywords:** Epidemiology, overactive bladder

## Abstract

Although it is suggested that in the United States overactive bladder affects one out of six individuals, this estimation may represent a subset of the population. Using a Pubmed literature search, many studies do not address those in a lower socioeconomic strata and the prevalence of overactive bladder may be higher. Overactive bladder symptoms may be under-reported in this population due to social stigma, lack of education or inaccessibility to medical care. This paper proposes to perform an epidemiological study incorporating validated incontinence questionnaires to assess the prevalence of overactive bladder symptoms among Indian women.

Overactive bladder (OAB) is a clinical problem of significant magnitude.[1,2] Limited data exists on its prevalence in the minority women or in those of lower socioeconomic status.[3,4] A previous study did not analyze minority subsets while others utilized non validated questionnaires or retrospective data.[1,5,6] This prospective study aims to determine the prevalence of OAB symptoms with and without urge incontinence in women of lower socioeconomic strata. A working hypothesis is that the prevalence of OAB may be high in such a population as it could be under-reported by patients. This may be due to lack of education, economic hardships or social stigma. If a high prevalence rate is encountered, this population should benefit greatly from education and treatment.

## DESCRIPTION OF STUDY

The study should be multi-institutional in scope. It should consist of two stages: Phase 1 should include initial screening with a modified validated questionnaire while Phase 2 should consist of diagnostic testing for OAB in those with a positive questionnaire. Testing should involve urine culture and cytology, voiding diaries and urodynamic studies. Intervention consisting of behavior modification and possible drug therapy is the goal.

A women's clinic or hospital averaging approximately 100 outpatient visits daily can be a site for the study. The study should be conducted in outpatient clinics over a six-month period. The study population should consist of women ≥ 18 years of age. Exclusion criteria include current pregnancy, women who have delivered within three months and those diagnosed with active urinary tract infection.

After obtaining Institutional Review Board approval, a confidential self-administered questionnaire incorporating the short forms of the Incontinence Impact Questionnaire (IIQ-7) and the Urogenital Distress Inventory (UDI-6) should be provided in the clinic. Additional pertinent clinical data including medical co-morbidities, current medications, previous pelvic surgery, obstetrical history, smoking history and BMI should be collected. Socioeconomic demographics should be assessed by asking patients to provide information on ethnicity, education level attained and annual income (see Appendix). Native language versions of the questionnaire should be available for those who cannot read Hindi or English. All responses should be held strictly confidential.

Response rate should be determined by the number of questionnaires collected divided by total clinic visits during the six-month study period. All data should be entered into a computer database program and statistical analysis performed. Significant trends and correlations should be identified and reported.

One potential problem may involve the selection process. As the study population is not randomly selected, there may be an effect on the study outcome. To minimize a poor response rate and to provide an incentive to complete the questionnaire, small items such as pens or educational paraphernalia should be supplied.

### Previous work

In 2003, the International Continence Society characterized OAB as urgency, with or without urge incontinence, which is often accompanied by increased micturition frequency (more than eight voids per day) and nocturia. These symptoms occur in the absence of identifiable etiologies such as urinary tract infection, interstitial cystitis or bladder cancer.[2,7]

In the United States, it is suggested that one in every six adults should be affected.[1] The National Overactive Bladder Evaluation (NOBLE) study further clarified the prevalence of OAB: the estimated prevalence is similar among men and women, 16% and 16.9%, respectively. In women, the prevalence of OAB with incontinence ranges from 2% (aged 18-24 years) to 19% (aged 65-74 years), increasing markedly after age 44 years.[1] Patients with OAB reported more health problems, including diabetes and congestive heart failure and had an average of 84% more yearly visits to a physician than those without OAB.[8] Postmenopausal women with urge incontinence have a substantially higher risk of falling and sustaining a fracture than women without urge incontinence.[9] The annual costs associated with OAB in the community setting are greater than $9 billion, including $2.9 billion for diagnosis and treatment, $1.5 billion for routine care, $3.9 billion for treatment of health-related consequences and $841 million in lost productivity.[10]

There are limited studies regarding the prevalence of OAB in minority populations. In a study by Bump,[11] 56% out of 54 black women described symptoms of urge incontinence. Sze *et al*[5] surveyed a total of 2370 patients, 34% black, 39% white and 27% Hispanic. The percentage of women who had urge incontinence symptoms was very similar between the three groups (19% vs. 16% vs. 16%, *P*=0.214). The study, however, did not use a validated questionnaire. Duong and Korn,[6] in a retrospective analysis, revealed that detrusor instability was diagnosed more often in black women than in Hispanic, white or Asian women (29% vs. 8%, 15% and 14%, respectively; *P*<0.001, *P*=0.04 and *P*=0.04). Another study evaluating the differences between white and Hispanic women showed the symptoms of urge incontinence in 18% of white women compared to 9% in Hispanic women (*P*= 0.019). The power was limited in this study, however, secondary to a small number of white women enrolled in the study.[12] In a community-based survey of elderly Mexican-American women, 15% of 1,589 reported having urinary incontinence. Of those incontinent patients, 33% reported urge incontinence and 15% reported symptoms severe enough to keep them from engaging in social activities.[13] An epidemiologic study of OAB with or without urinary incontinence in a cohort of younger Indian women does not exist.

This proposal should analyze the prevalence of OAB symptoms in a minority, lower socioeconomic female population. Using a questionnaire based on validated forms, this study should evaluate symptoms, patient demographics and medical co-morbidities. Our hypothesis is that OAB symptoms may be under-reported in such a population, because of lack of education, social stigma or economic hardships. If a high prevalence is encountered, intervention with behavior modification and drug therapy may be beneficial.

## Appendix

**Table:**

1)	Initials:					
2)	Age:					
3)	Ethinicity:					
4)	Number of normal deliveries:		C/sect.	Abortions	Miscarriage	
5)	Educational Level < High School		High School	Technical/ some college	College graduate	Unknown
6)	Household Income (please check)					
			<20, 000	20,000-29,000	30,000-$49,000	over 50,000
	I refuse to volunteer information					
7)	Please list any medical problems:					
8)	Have you had any previous surgery performed for incontinence or prolapse (“dropped bladder or uterus”)?					
9)	Were you recently diagnosed with a urinary tract infection in the past four weeks?					
			Y	N		
10)	Do you experience and, if so, how much are you bothered by:					
	a)	Frequent urination?				
		Not at all (0)	Slightly (1)	Moderately (2)	Greatly (3)	
	b)	Urine leakage related to the feeling of urgency?				
		Not at all (0)	Slightly (1)	Moderately (2)	Greatly (3)	
	c)	Urine leakage related to physical activity, coughing or sneezing?				
		Not at all (0)	Slightly (1)	Moderately (2)	Greatly (3)	
	d)	Small amounts of urine leakage (drops)?				
		Not at all (0)	Slightly (1)	Moderately (2)	Greatly (3)	
	e)	Difficulty emptying your bladder?				
		Not at all (0)	Slightly (1)	Moderately (2)	Greatly (3)	
	f)	Pain or discomfort in the lower abdominal or genital area?				
		Not at all (0)	Slightly (1)	Moderately (2)	Greatly (3)	
11)	Has urine leakage and/or prolapse affected your:					
	a)	Ability to do household chores (cooking, housecleaning, laundry)?				
		Not at all (0)	Slightly (1)	Moderately (2)	Greatly (3)	
	b)	Physical recreation such as walking, swimming or other exercise?				
		Not at all (0)	Slightly (1)	Moderately (2)	Greatly (3)	
	c)	Entertainment activities (movies, concerts, etc.)?				
		Not at all (0)	Slightly (1)	Moderately (2)	Greatly (3)	
	d)	Ability to travel by car or bus more than 30 minutes from home?				
		Not at all (0)	Slightly (1)	Moderately (2)	Greatly (3)	
	e)	Participation in social activities outside your home?			
		Not at all (0)	Slightly (1)	Moderately (2)	Greatly (3)	
	f)	Emotional health (nervousness, depression, etc.)?				
		Not at all (0)	Slightly (1)	Moderately (2)	Greatly (3)	
	g)	Feeling frustrated?				
		Not at all (0)	Slightly (1)	Moderately (2)	Greatly (3)	
12)	Have you ever discussed your urinary problems with your doctor?					
	Y		N			
	If no, why not?					
13)	Has the issue of urinary leakage or urinary frequency ever been discussed by your Doctor?					
	Y		N			
14)	Do you use any pads or protective garment?					
	Y		N			
15)	Do you smoke? If so, how many cigarettes per day?					
	Y		N			
16)	How many caffeinated drinks (tea, coffee, soda) do you have per day?					
17)	Do you drink alcohol?					
	Not at all		Occasionally	Moderately	Greatly	
18)	What is your height?					
19)	What is your weight?					
